# Tic Reduction in Adult Onset Gilles De La Tourette Syndrome Using as Required Nabiximols Spray

**DOI:** 10.5334/tohm.613

**Published:** 2021-08-06

**Authors:** Maximilian A. Schwittay, Andreas Steinbrecher, Elmar Lobsien

**Affiliations:** 1 Department of Neurology, Helios Klinikum Erfurt, DE; 2 Department of Psychiatry and Psychotherapy, Sophien- und Hufeland-Klinikum Weimar, DE

**Keywords:** Tourette, Tic, Cannabinoids, Neuropharmacology, hyperkinetic movement disorder, neurodevelopmental disorder, on demand treatment

## Abstract

**Background:**

Tourette syndrome (TS) manifests with motor and vocal tics that can reach disabling intensity. Established therapies may show insufficient relief or side effects. Cannabinoids have demonstrated therapeutic potential in small studies. This report presents buccal Nabiximols “as required” in the treatment of tics in TS.

**Case report:**

A 25-year-old man presented with stigmatizing motor and phonic tics after cessation of daily Cannabis use. After Tiaprid 300 mg per day had shown no sufficient effect a trial of Nabiximols reduced tics by >90%.

**Discussion:**

Nabiximols could be an adjunct treatment in TS for situations were tics are severly disabling.

## Background

Gilles de la Tourette syndrome (TS) is a chronic neurodevelopmental disorder which manifests as multiple simple or complex motor and vocal tics with accompanying psychiatric phenomena. A great variation in the clinical spectrum as well as in the intensity and frequency of tics is common. Disease usually begins in childhood and peaks during adolescence, but late- or adult-onset variants are also recognised [1]. Males are more commonly affected, the general prevalance is approximated at below 1%. Established treatments include behavioral (Comprehensive Behavioral Intervention [CBIT], Habit Reversal [HRT]) and pharmacological (neuroleptics & anti-dopaminergics) therapies. More recently advanced surgical techniques like deep brain stimulation (DBS) were shown to be effective in a subgroup of very severely affected and otherwise treatment-refractory cases [2]. Despite these therapeutic advances a relevant number of treated patients suffer from insufficient symptom reduction or unwanted side effects of medications. Additionally, access to behavioral therapies is limited by the avaibility of specialised therapists. Therefore there is a demand for well-tolerated, broadly avaible and effective treatments [2,3]. Cannabinoids have repeatedly demonstrated their therapeutic potential in case reports and small studies since 1988 [4,5,6]. Commonly used agents of medical cannabis and Cannabis-based medicine (CBM) contain different proportions and preparations of Cannabidiol (CBD) and Tetrahydrocannabinol (THC). We describe here the “as required” use and effect of Nabiximols, which consists of a combination of both [7] and has already been shown to be effective when used as a longer-term-medication in case reports [5,7]. Treatment in Germany, as well as in many other countries, is an “off label” application and depends on insurance company cost-overage and a permission of the responsible federal department [8].

## Case report

A 25 year old man presented with a one week history of rapidly progressive simple and complex motor (e.g. nodding, pointing, head shaking and turning, looking, blinking, hitting) and vocal (e.g. shouting, humming) tics including motor and vocal echo and possible blocking phenomena, all of which he attributed to the cessation of his recreational use of cannabis a few weeks ago. For at least one year he had consumed daily to relief the “urge to move the legs in the evening” that started two years ago. Back then he first noticed short twitching, disturbing movements in his legs at night, later during the daytime as well. For several years before that, he had experienced episodes of diffuse urgency to move his legs but had never had any tics or other visible unconsciuos movements. Suppressing the movement had become more difficult in the last weeks, making leaving his home or usage of public transport increasingly stigmatizing, resulting in self isolation. At the time of presentation he had continuous tics, consisting mainly of head and upper extremity movements, which he could suppress for up to 10 seconds. Exacerbating factors included emotional distress and being exposed to repetitive sounds or movements. Pre-treatment severity was determined at 75/100 measured on the Global score of the Yale Global Tic Severity Scale (YGTSS-GS). Apart from a single depressive episode several years ago treated with escitalopram and labelling himself as a demanding child he had nil else of note in his medical history and he was not on any medication. Family history was negative except for the father’s habit “to clear his throat quite often”. He appeared fit and well and general examination was normal. Neurological examination revealed no further pathological signs and psychiatric assessment was not suggestive of relevant cognitive or psychiatric comorbidities besides the current social isolation. Baseline laboratory tests including differential, serum copper indices and antibodies (NMDA, AMPA-A/B, GABA1, VGCC, LGI1, CASPR2, GAD, CV2, Hu, Ri) were within normal ranges. CSF examination, chest radiography and abdominal ultrasound were normal. MRI of the brain showed no lesions and was age-appropriate. In summary, we supected an late-onset TS, exacerbated after stopping self-treatment with cannabis. An adult-onset secondary, or transient tic disorder could not be fully ruled out. Tiaprid 300 mg per day was administered but showed no sufficient symptom relief. Owing to the therapeutic benefit of cannabis for his symptoms together with their relatively acute onset following cessation we considered a trial of Nabiximols (Sativex®) spray. Buccal application of three doses in 15 min. intervals resulted in a dramatic decrease of symptom expression. Premonitory feeling vanished and intensity of motor and phonic tics were reduced clearly (YGTSS-GS 5/100), described as “>90%” by the patient (see ***video***). The effect lasted about 4 hours before tics started to return. Our patient reported no relevant side effects, besides cheerfullness and a feeling of relaxation.

**Supporting information:** The video shows a typical manifestation of tics during simple motor tasks (e.g. Finger-to-nose-test, holding arms straight) and after willingly suppressing tics for a few seconds. In the second section the video shows the therapeutic result at 15 min. after 3 puffs of Nabiximols while the patient reports about the effect of tiaprid (“… was suppressing the tics like holding them in a cage …”).

## Discussion

Regarding tics and associated disorders a reduction of symptom burden by Cannabis-based medicine has been shown in small studies and case reports, with self treatment appearing to be common. However, because of the sparcity of studies of older and newer Cannabinoids and results of the last and largest study “CANNA-TICS” still pending, until now no high quality evidence based recommendations could be made [9,10,11].

We chose Nabiximols because our patient described good effects in self treatment, affirmative results in other case reports, and the combination of THC and CBD being considered to reduce the undesirable effects of THC and allowing to use the CBD specific effects as well [5,7,11,12].

Nabiximols is a tincture of plant extracts with a defined THC:CBD-ratio including minor quantities of other plant derivates. Application is by buccal spray, one dose releasing 2.7 mg THC and 2.5 mg CBD. Pharmacokinetics vary interpersonally but the effect is seen for 2–4 hours starting just minutes after application. Up to 12 puffs per day can be used [13].

In comparison with the commonly used group of neuroleptics, which pose risks of extrapyramidal disturbances, tardive dyskinesia, weight change, sedation and QTC prolongation, the side effects of Nabiximols are supposed to be less severe, being mainly dizziness, dry mouth and fatigue. There is a wide variety of other possible Cannabis- or THC-associated side effects, mostly reported in recreational users, including the risk of psychosis in predisposed individuals. Medical application in the neurological context, which has been described for e.g. nystagmus, bladder dysfunction, spastiticy, epilepsy, dystonia, and headache, is estimated to pose minor risks through standardised dosing, application and preparations [14].

Most probably THC and CDB develop their effects through the central part of the Endocannabinoid system whose functions as a part of the general nervous, humoral and immune systems are still incompletely understood. It has been found to be involved in the regulation of complex physiologic processes including neuronal plasticity, control of movement or psychomotor behavior and, besides many others, immunity, learning and memory. Generators of those effects are in particular two receptors, called CB1 (cannabinoid receptor 1) and CB2, that modulate neurotransmitter release via G-Protein coupled pathways. If activated, an opening of potassium and closure of calcium channels results which inhibits the release of a selection of neurotransmitters, e.g. glutamate, GABA, serotonin, dopamine, noradrenaline and acetylcholine, making them possible “circuit breakers” on affected inhibitory and excitatory neurons. CB1, being the main actor of THC effects, is found mainly in the nervous system, especially centrally on efferent basal ganglia, cerebellar, and hippocampal neurons and the dorsal roots or afferent tracts of the spinal cord in close relation to dopaminergic neurons, supporting the supposed role in the plasticity and control of movements [14].

Our case underpins the results of two previous reports showing comparable longer-term reductions of tic severity using Nabiximols in a fixed regimen over 14 days with 2–3 sprays twice to thrice a day, slowly increasing the initial dosage [5,7]. Our patient, who knew the effect of his recreational use of cannabis had a marked tic reduction after 2–3 sprays lasting up to four hours without experiencing relevant side effects. This emphasizes, in our opinion, the potential of Nabiximols being used not only within a fixed drug regimen but as an easy to use and effective “acute” medication.

## Conclusion

Based on our case, and in line with previous reports, we propose that buccal Nabiximols might be an effective addition to “acute” or “as required” tic treatment under specialist guidance, especially for predictable situations in the short term when severly disabling or stigmatizing tics are anticipated.
